# Ginsenoside Rg3 induces mesangial cells proliferation and attenuates apoptosis by miR-216a-5p/MAPK pathway in diabetic kidney disease

**DOI:** 10.18632/aging.205907

**Published:** 2024-06-07

**Authors:** Yuanzhen Chen, Yuhuan Peng, Ping Li, Ying Jiang, Dan Song

**Affiliations:** 1 Department of Nephrology, Shenzhen Guangming District People’s Hospital, Guangming, Shenzhen 518000, China; 2 Department of Pharmacy, Shenzhen Guangming District People’s Hospital, Guangming, Shenzhen 518000, China

**Keywords:** ginsenoside Rg3, diabetic nephropathy, miR-216a-5p, MAPK pathway, apoptosis

## Abstract

Background: Ginsenoside Rg3 is an active saponin isolated from ginseng, which can reduce renal inflammation. However, the role and mechanism of Rg3 in diabetic kidney disease (DKD) are far from being studied.

Methods: The effects of Rg3 and miR-216a-5p on the proliferation, apoptosis, and MAPK pathway in high glucose (HG)-induced SV40 MES 13 were monitored by CCK-8, TUNEL staining, and western blot.

Results: Rg3 treatment could accelerate proliferation and suppress apoptosis in HG-induced SV40 MES. Moreover, miR-216a-5p inhibition also could alleviate renal injury, prevent apoptosis, and activate the MAPK pathway in kidney tissues of diabetic model mice.

Conclusion: Rg3 could attenuate DKD progression by downregulating miR-216a-5p, suggesting Rg3 and miR-216a-5p might be the potential drug and molecular targets for DKD therapy.

## INTRODUCTION

MicroRNA (miRNA) is a non-coding small molecule RNA of 21–24 nucleotides in length that can bind to mRNA through base complementary pairing to control the translation or degradation of the target mRNA [1]. Several studies have manifested that miRNAs can prevent or accelerate Diabetic kidney disease (DKD) progressions [2, 3]. miR-216a-5p is a member of the miRNA family, can attenuate the process of various diseases, such as cancer [4, 5], Alzheimer’s disease [6], bronchopneumonia [7], neuropathic pain [8], etc. Additionally, research indicated that miR-216a-5p could induce HG-induced EMT and fibrogenesis by BMP7 in DKD [9]. However, the impact of miR-216a-5p on mesangial cell apoptosis remains unclear.

DKD as one of the major microvascular complications of diabetes, is now one of the leading causes of death in patients with end-stage renal disease (ESRD) [10]. Diabetes can cause multiple pathological structural changes in the kidney, such as vascular endothelial dysfunction, glomerular enlargement, thickening of the basement membrane, mesenchymal proliferation, and increased extracellular matrix [11, 12]. The clinical manifestations of DKD are the persistent decline of renal function, albuminuria, and edema [13]. Without intervention, it can progress to glomerulosclerosis, eventually leading to renal failure [14]. The occurrence of DKD is a long pathological process with complex mechanisms. Numerous clinical and experimental studies have suggested that inflammation is a key factor in the deterioration of renal function [15]. In the state of hyperglycemia, the inflammation of renal tissue is initiated through multiple signal pathways, among which the MAPK pathway is the most classical inflammatory pathway [16]. After activation, the MAPK pathway can phosphorylate different transcription factors, which can result in the production of inflammatory factors and participate in the inflammatory response of diseases [17]. Therefore, it is vital to search for novel drugs and molecular targets that can block the activation of the MAPK pathway for DKD therapy.

Based on the characteristics of clinical manifestations, DKD can be classified into the categories of traditional Chinese medicine (TCM) “edema”, “deficiency”, and “Guange” [18]. It is mainly caused by spleen and kidney deficiency, dampness, and blood stasis block and is treated by invigorating the spleen and kidney and eliminating the turbid by purgation [19]. Ginseng is a rare TCM with a long history. It has the functions of enriching yin and nourishing kidney, invigorating spleen for benefiting lung, calming mind and enhancing intelligence [20, 21]. Ginsenoside is the main active component of ginseng, which also has anti-inflammatory, anti-microbial, antibacterial, anti-parasitic, anti-tumor, anti-virus, and other effects [22]. Ginsenoside Rg3 is a saponin compound in the TCM ginseng, which has a series of pharmacological activities such as alleviating inflammatory response, reducing oxygen free radicals, and anti-oxidation [23–25]. Studies manifested that Rg3 can alleviate the progression of DKD by enhancing antioxidant capacity, reducing inflammation, and anti-apoptosis [26, 27]. Rg3 can also attenuate high glucose (HG)-induced mesangial cell proliferation and inflammation by inhibiting NF-κB [26]. Besides, Research indicated that Rg3 can induce activated receptors and increase the activity of natural killer cells by the MAPK pathway [28]. Rg3 can restrain the proliferation of melanoma cells by weakening the MAPK pathway [29]. Rg3 can also induce angiogenesis in human endothelial cells through MAPK/ERK pathway [30]. These data suggested that Rg3 can induce the inactivation of the MAPK pathway. Therefore, further investigation of the effects of Rg3 on the MAPK pathway, renal histopathological injury, and apoptosis may provide a new experimental basis for the protective mechanism of Rg3 in renal tissue injury.

In our study, we constructed diabetic cell and mouse models and explored the influences of Rg3 on the proliferation and apoptosis in HG-treated SV40 MES and kidney tissues of diabetic model mice. Besides, we also explored the possible gene regulated by Rg3 in DKD progression and its effect on the MAPK pathway. Therefore, this study may provide a theoretical basis for the mechanism and clinical application of Rg3 in DKD.

## MATERIALS AND METHODS

### Cell culture, construction of diabetic model cells, and treatment

Mouse mesangial cells (SV40 MES 13) were purchased from ATCC (USA) and incubated in DMEM (Gibco, USA) containing 10% fetal bovine serum (FBS, Gibco) and low sugar (5.5 mM) at 37°C and 5% CO_2_. Normal-cultured SV40 MES cells were disposed of with increasing concentrations of Rg3 (1, 2, 4, 6, 8, 16, and 32 μM) for 48 h. To obtain diabetes model cells, SV40 MES 13 were incubated with DMEM including 30 mM glucose for 48 h [31]. miR-216a-5p mimics, miR-216a-5p inhibitors, and corresponding negative controls (NC) were acquired from GenePharma (Suzhou, China). And the high glucose (HG)-treated SV40 MES were treated with 2, 4, and 8 μM Rg3 for 48 h. Besides, HG-treated SV40 MES were addressed with 8 μM Rg3 and transfected with the miR-216a-5p mimics, inhibitor, or NC using Lipofectamine™ 3000 (Invitrogen, USA) for 48 h.

### Cell viability analysis

SV40 MES were inoculated into 96-well plates with 2 × 10^4^ cells/well and treated based on the research design. Then 10 μl CCK-8 was added to each well and incubated for 2 h away from light. Absorbance at 450 nm was examined with a microplate reader, which was applied to evaluate the proliferation capacity.

### TUNEL staining

For cells, groups of SV40 MES were made into cell smears. After fixation with 4% paraformaldehyde (Sigma-Aldrich, USA), TUNEL staining and DAPI staining were performed. And the apoptotic cells were observed by a fluorescent microscope (Olympus IX71). For tissues, the kidney tissues mice were made into paraffin sections (3 μm). Then the sections were dewaxed, dehydrated, dried, processed with trypsin K, added with the reaction solution, and incubated in a thermostat at 37°C for 1 h. After washing, the sections were stained with DAB, re-stained with hematoxylin, dehydrated with 75%, 80%, 95%, and 100% ethanol, and transparent with xylene. After sealing the sections with neutral gum, the sections were placed under a microscope for observation.

### Western blot

Total protein was extracted with RAPI lysate (Beyotime, China) from SV40 MES 13 or mouse kidney tissues, and the concentration was confirmed using a BCA kit. 30 μg equal samples were separated by SDS-PAGE, then transferred to PVDF membrane (Millipore, USA), enclosed in 5% skim milk for 2 h. Then the membranes were exposed to primary antibodies including cle-Caspase 3 (Abcam, 1: 1000), cle-Caspase 8 (Abcam, 1: 500), Bcl2 (Abcam, 1: 500), Bax (Abcam, 1: 500), p-p70s6k (Abcam, 1: 500), p70s6k (Abcam, 1: 1000), p-MAPK (Abcam, 1: 500), MAPK (Abcam, 1: 1000) or GAPDH (Abcam, 1: 2000) at 4°C overnight and secondary antibody for 2 h. After washing, the membrane was treated with ECL reagent (Millipore) and developed. Protein bands were analyzed by a Gel imaging System ((Bio-Rad, USA). The gray value was analyzed using ImageJ Software. GAPDH served as a loading control.

### qRT-PCR

Total RNA was acquired from mouse kidney tissues or SV40 MES 13 by TRIzol (Invitrogen, USA). After the purity and concentration of RNA were qualified, cDNA was synthesized using a reverse transcription kit (Takara, Tokyo, Japan) based on the instructions. SYBR Green qPCR Master Mix (DBI Bioscience, China) was applied for the qRT-PCR analysis. And the level of miR-216a-5p was calculated by 2^−ΔΔCt^.

### Construction of diabetic model mice

Healthy SPF C57BL/6 mice (male, aged 6–7 weeks, weight 21–22 g) were purchased from Experimental Animal Center and fed in an SPF barrier system for one week at a constant temperature and given a 12:12 h circadian cycle of day and night, as well as clean food and drinking water. Then mice were randomly divided into a control group (*n* = 6) and a model group (*n* = 18). Mice in the model group were intraperitoneally injected with 50 mg/kg STZ (STZ dissolved in 0.1 mol/L sodium citrate buffer, pH4.5) for 5 consecutive days to establish a diabetes mouse model. The mice in the control group accepted the same volume of citrate buffer. Fasting blood glucose was measured by blood glucose meter after 7 d of modeling, and mice with blood glucose >16.7 mMafter fasting were considered to be successfully modeled. The modeled mice were divided into 3 groups: diabetes model group (DKD, *n* = 6), Rg3 administration group (DKD+Rg3, the mice were treated by oral gavage of the 20 mg/kg Rg3 for 8 weeks, *n* = 6), and miR-216a-5p inhibitor group (DKD+inhibitor, the mice were injected with miR-216a-5p inhibitors through a tail vein for 3 days, *n* = 6). The protocol was approved by the research ethics committees of the Shenzhen Guangming District People’s Hospital (approval no. of ethics committee: LL-KT-2020075).

### H&E staining

Kidney tissue was fixed with 4% paraformaldehyde for 72 h, dehydrated by alcohol gradient, and embedded. Then the sections were cut into 4 μm by pathological microtome. After dewaxing and hydration, the sections were stained with H&E and sealed with neutral gum. The histological manifestation was observed with a microscope.

### Statistical analysis

All data were expressed as mean ± SD and analyzed by SPSS26.0 software with one-way ANOVA. *P* < 0.05 was considered statistically significant.

## RESULTS

### Determination of Rg3 concentration in SV40 MES

**Figure 1:**
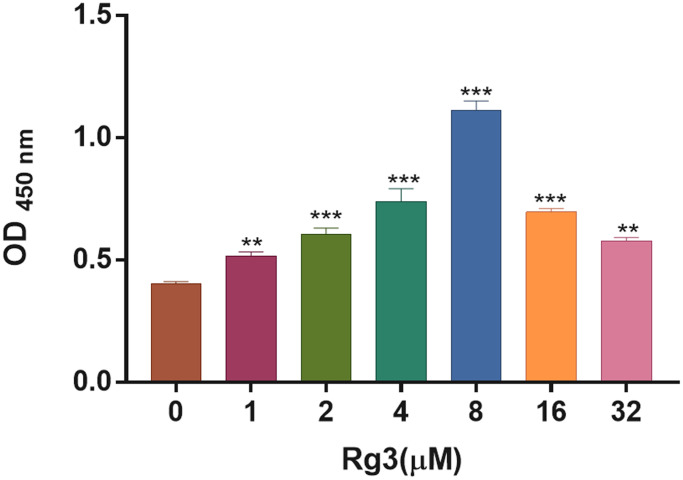
**Screening of appropriate Rg3 concentration in SV40 MES 13.** CCK-8 was conducted to confirm the change of cell proliferation in SV40 MES 13 after treating with 1, 2, 4, 8, 16, and 32 μM Rg3. ^**^P < 0.01, ^***^P < 0.001.

To confirm the optimal concentration of Rg3 for treating SV40 MES. We applied different concentration gradients (0, 1, 2, 4, 8, 16, and 32 μM) of Rg3 to treat SV40 MES for 24 h. And the CCK-8 results demonstrated that the OD value in Rg3-treated SV40 MES 13 was notably (*p* < 0.001) increased compared with the control group, and the proliferation capacity (OD value) of SV40 MES reached the peak when Rg3 concentration was 8 μM (Figure 1). Thus, 8 μM Rg3 was considered the optimal concentration for SV40 MES treatment.

### Rg3 treatment induces proliferation and prevents apoptosis in HG-induced SV40 MES

**Figure 2:**
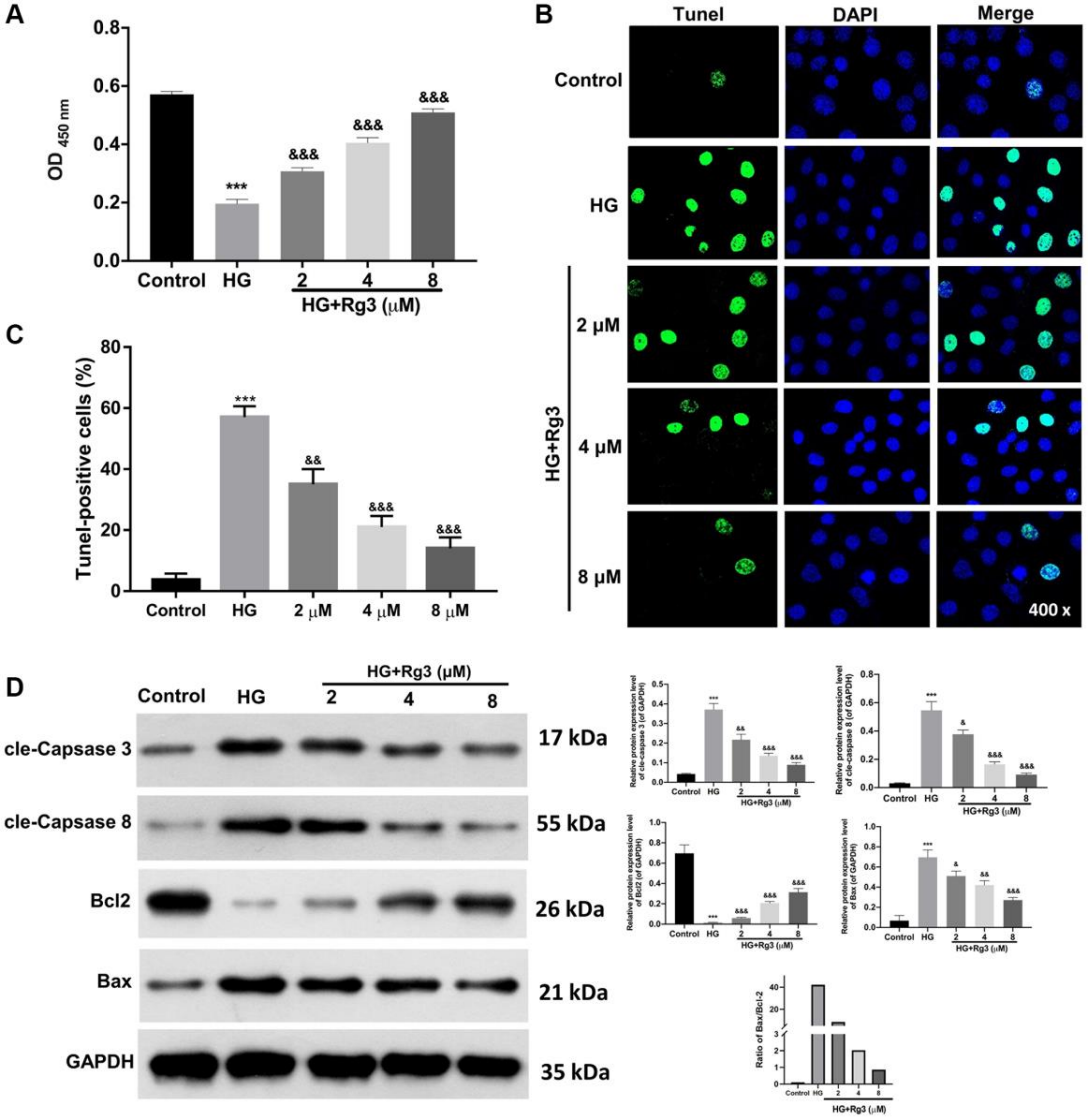
**Rg3 induces proliferation and prevents apoptosis in HG-induced SV40 MES 13.** HG-treated SV40 MES 13 were processed with 2, 4, and 8 μM Rg3. (**A**) Cell proliferation was monitored via CCK-8 in each group. (**B**) TUNEL analysis of cell apoptosis (Magnification, 200×). (**C**) Quantitative analysis of TUNEL results. (**D**) Western blot denoted the changes of cle-Caspase 3, cle-Caspase 8, Bcl2, and Bax expressions. ^***^P < 0.001 vs. control group; ^&&^*P* < 0.01 and ^&&&^*P <* 0.001 vs. HG group.

To investigate the mechanism of Rg3 in DKD, we constructed the DKD model of SV40 MES through 30 mM of HG treatment for 48 h. Then the HG-induced SV40 MES were addressed with 2, 4, and 8 μM Rg3. CCK-8 results suggested that the proliferation ability of SV40 MES 13 was markedly (*p* < 0.001) reduced in the HG group relative to that in a control group, while Rg3 treatment could markedly improve the proliferation capacity of HG-induced SV40 MES 13 (Figure 2A). Meanwhile, the TUNEL data indicated that HG-induced apoptosis could be memorably (*p* < 0.001) reversed after administration with Rg3 in SV40 MES 13 (Figure 2B, 2C). It was well known that up-regulation of cle-Caspase 3, cle-Caspase 8, and Bax expressions and downregulation of Bcl2 could expedite apoptosis. And our results exhibited that cle-Caspase 3, cle-Caspase 8, and Bax were distinctly (*p* < 0.001) up-regulated, and Bcl2 was signally (*p* < 0.001) decreased in HG-induced SV40 MES 13 relative to the control cells, while Rg3 could markedly reverse the expressions of these proteins in HG-induced SV40 MES 13 (Figure 2D).

### Rg3 induces proliferation, suppresses apoptosis, and activates the MAPK pathway by downregulating miR-216a-5p in HG-induced SV40 MES 13

**Figure 3:**
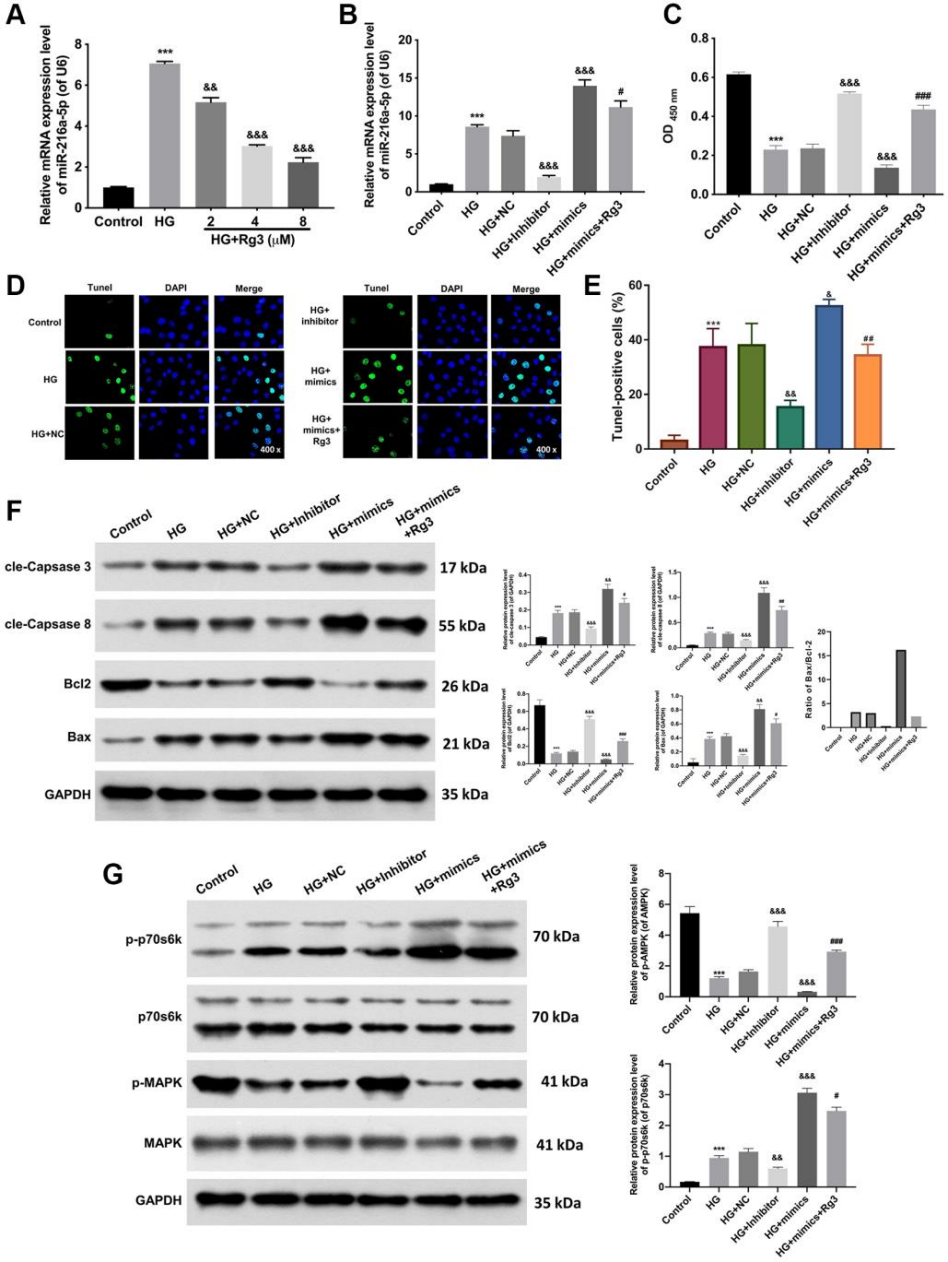
**Rg3 enhances proliferation, attenuates apoptosis, and activates the MAPK pathway through the downregulation of miR-216a-5p in HG-induced SV40 MES 13.** (**A**) qRT-PCR analysis for the evaluation of a miR-216a-5p expression in HG-induced SV40 MES 13 after processing with Rg3. (**B**) HG-induced SV40 MES 13 were transfected with miR-216a-5p inhibitor, miR-216a-5p mimics or NC, and treated with Rg3. The expression change of miR-216a-5p was identified by applying qRT-PCR. (**C**) CCK-8 assay for the examination of cell proliferation in SV40 MES 13 treated in the same way as B. (**D**) TUNEL staining revealed the change of cell apoptosis (Magnification, 200×). (**E**) TUNEL-positive cells were quantified. (**F**) The changes of cle-Caspase 3, cle-Caspase 8, Bcl2, and Bax expressions were confirmed through the application of western blot. (**G**) Western blot analysis of p-p70s6k, p70s6k, P-MAPK, and MAPK expressions in each group. ^***^P < 0.001 vs. control group; ^&&^*P* < 0.01, ^&&&^*P* < 0.001 vs. HG + NC group; ^#^*P* < 0.05, ^##^*P* < 0.01, ^###^*P* < 0.001 vs. HG + mimics group.

Through literature analysis, we found that miR-216a-5p was highly expressed in HG-treated kidney cells [9]. And we also confirmed that miR-216a-5p expression was aggrandized in HG-induced SV40 MES 13, while Rg3 treatment dramatically (*p* < 0.001) reversed the upregulation of miR-216a-5p mediated by HG induction (Figure 3A). To further certify the mechanism of Rg3 and miR-216a-5p in DKD. HG-induced SV40 MES 13 cells were treated with miR-216a-5p inhibitors, mimics, and 8 μM Rg3. qRT-PCR results revealed that miR-216a-5p was upregulated (*p* < 0.001) in HG-induced SV40 MES 13, and miR-216a-5p inhibition downregulated miR-216a-5p, miR-216a-5p overexpression upregulated miR-216a-5p in HG-induced SV40 MES 13, and Rg3 also could reduce the upregulation of miR-216a-5p mediated by miR-216a-5p overexpression in HG-induced SV40 MES 13 (Figure 3B). Thus, we verified the successful transfection of miR-216a-5p inhibitors and miR-216a-5p mimics. Besides, CCK-8 displayed that inhibition of miR-216a-5p conspicuously (*p* < 0.001) strengthened proliferation, overexpression of miR-216a-5p strikingly (*p* < 0.001) weakened proliferation in HG-induced SV40 MES 13, while Rg3 notably (*p* < 0.001) alleviated the attenuated effect of miR-216a-5p overexpression on cell proliferation in HG-induced SV40 MES 13 (Figure 3C). And TUNEL data revealed that miR-216a-5p inhibition distinctly restrained apoptosis and miR-216a-5p overexpression outstandingly (*p* < 0.001) activated apoptosis, while Rg3 observably (*p* < 0.001) attenuated the promoting effect of miR-216a-5p overexpression on the apoptosis of HG-induced SV40 MES 13 (Figure 3D, 3E). Moreover, western blot results presented that miR-216a-5p inhibition notably (*p* < 0.001) downregulated cle-Caspase 3, cle-Caspase 8, and Bax, and upregulated (*p* < 0.001) Bcl2 in HG-treated SV40 MES 13, and the effect of miR-216a-5p overexpression on these proteins was opposite to miR-216a-5p inhibition, and Rg3 also could outstandingly (*p* < 0.001) reverse the upregulation of cle-Caspase 3, cle-Caspase 8, and Bax, and the downregulation of Bcl2 mediated by miR-216a-5p overexpression in HG-treated SV40 MES 13 (Figure 3F). Meanwhile, our data revealed that inhibition of miR-216a-5p memorably (*p* < 0.001) downregulated p-p70s6k and upregulated p-MARK, and overexpression of miR-216a-5p prominently (*p* < 0.001) upregulated p-p70s6k and downregulated p-MARK in HG-induced SV40 MES 13, while Rg3 also could markedly (*p* < 0.001) attenuate the expression changes in p-p70s6k and p-MARK proteins mediated by miR-216a-5p overexpression in HG-treated SV40 MES 13 (Figure 3G).

### Rg3 or miR-216a-5p inhibition ameliorates renal pathology, prevents apoptosis, and induces MAPK pathway in kidney tissues of diabetic model mice

**Figure 4:**
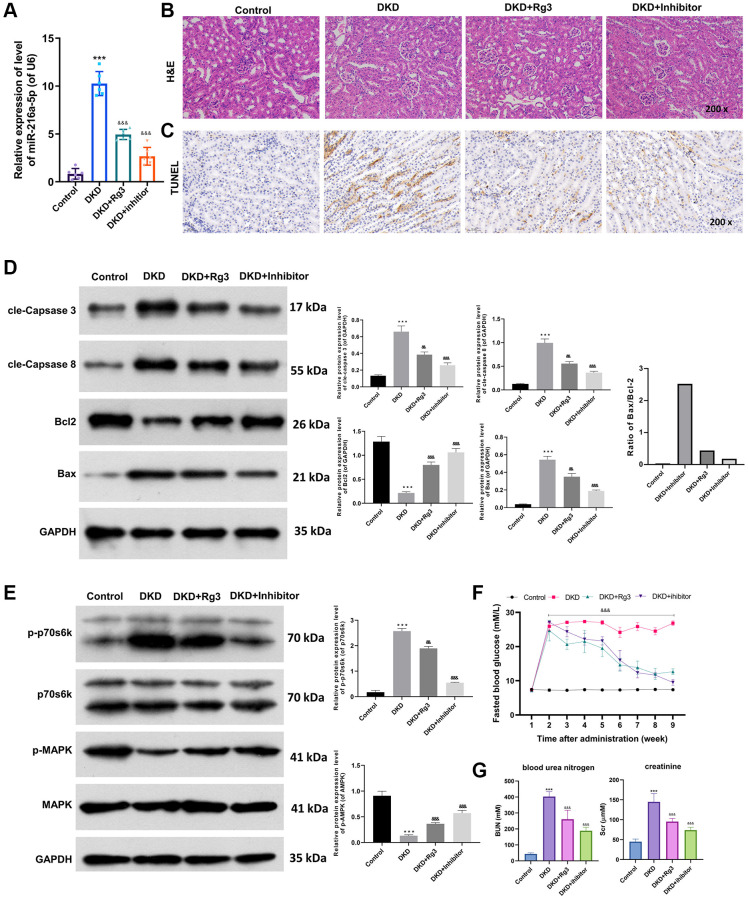
**Rg3 or miR-216a-5p inhibition alleviates kidney injury, prevents apoptosis, and induces MAPK pathway in kidney tissues of diabetic model mice.** And we first fed mice with STZ to build a mouse diabetes model, and then treated by oral gavage of the 20 mg/kg Rg3 for 8 weeks or injected with miR-216a-5p inhibitors through a tail vein (once a day for 3 days). (**A**) qRT-PCR was adopted to assess the change of miR-216a-5p in the mice kidney tissues. (**B**) H&E staining indicated the pathological change of kidney tissues (Magnification, 200×). (**C**) TUNEL staining for the confirmation of cell apoptosis (Magnification, 200×). (**D**) Western blot was applied to analyze the expression changes of apoptosis-related proteins. (**E**) Western blot showed the changes of p-p70s6k, p70s6k, p-MAPK, and MAPK expressions. (**F**) Fasting blood glucose. (**G**) Blood urea nitrogen and creatinine. ^***^P < 0.001 vs. control group; ^&&^*P* < 0.01, ^&&&^*P* < 0.001 vs. DKD group.

Moreover, we further assessed the mechanism of Rg3 in DKD through *in vivo* experiment. And a mouse diabetes model was built by feeding mice with STZ, and then the model mice were intragastric treated with 20 mg/kg Rg3 or injected with miR-216a-5p inhibitors. And the renal tissue was collected for a follow-up examination. qRT-PCR data revealed that miR-216a-5p was upregulated (*p* < 0.001) in the renal tissues of DKD model mice relative to that in control mice, while Rg3 or miR-216a-5p inhibition prominently (*p* < 0.001) attenuated the upregulation of miR-216a-5p in DKD model mice, especially miR-216a-5p inhibition (Figure 4A). And H&E staining results signified that in the control group, renal tissues were neatly aligned; in the DKD group, renal tissues showed typical glomerular injury, such as glomerulosclerosis, cellular vacuolization, basement membrane hyperplasia, and inflammatory cell infiltration; after Rg3 or miR-216a-5p inhibitor intervention, renal injury damage and pro-inflammatory cell infiltration were reduced, and there was some degree of repair of the renal basement membrane (Figure 4B). Similarly, TUNEL results exhibited that the apoptotic capacity of renal tissues was markedly increased in DKD model mice, which also could be memorably attenuated by Rg3 treatment or miR-216a-5p inhibitor (Figure 4C). Besides, western blot results displayed that cle-Caspase 3, cle-Caspase 8 and Bax were conspicuously (*p* < 0.001) upregulated, and Bcl2 was downregulated (*p* < 0.001) in the renal tissues of DKD model mice, which also could be dramatically reversed by Rg3 treatment or miR-216a-5p inhibition, especially miR-216a-5p inhibition (Figure 4D). Then we also disclosed that Rg3 or miR-216a-5p inhibition could obviously (*p* < 0.001) weakened the upregulation of p-p70s6k and downregulation of p-MARK in the renal tissues of DKD model mice, and the weakening effect of miR-216a-5p inhibition was more obvious (*p* < 0.001) than that of Rg3 (Figure 4E). The blood glucose was measured (Figure 4F). Compared with DKD group, FBG levels were (*p* < 0.001) significantly decreased in DKD+Rg3 and DKD+inhibitor group since the third week and continued to the end of this research. Renal measurements including blood urea nitrogen (BUN) and creatinine (Scr) were measured and shown in Figure 4G. We found that the levels of BUN and Scr were decreased significantly (*p* < 0.001) in Rg3 and inhibitor treatment when compared with DKD group.

## DISCUSSION

The World Health Organization predicts that the number of people with diabetes will increase to 300 million worldwide by 2025 [32]. As the familiar complication of diabetes mellitus, DKD has become a crucial cause of chronic renal failure in the elderly [33]. The pathogenesis of DKD is complex and has not yet been fully understood. While studies have testified that metabolic disorders caused by persistent hyperglycemia are the leading cause of DKD [34]. Currently, Chinese herbs (astragalus, ginseng, and coptidis rhizome) have good hypoglycemic efficacy, and exhibit unique advantages in blood glucose control, such as multi-function, multi-target, low toxicity and side effects, safety and reliability, mild and long-lasting effects, which can effectively slow down the occurrence of DKD [35–37]. Ginseng, as a TCM, is useful in the prevention and therapy of numerous diseases. Among them, ginsenoside Rg3 is mainly discovered in the root of ginseng, which belongs to the phytosterols of natural products. Rg3 has various pharmacological effects, such as anti-tumor, anti-cardiovascular disease, antidepressant, anti-inflammatory, and neuroprotection, and can regulate multiple signal transduction pathways and molecular targets [24, 38]. In the streptozotocin (STZ) -induced DKD rat model, Rg3 was also proved to protect the kidney by reducing oxidative stress and apoptosis [26, 27]. In this study, we also revealed that Rg3 has an inducing effect on proliferation and a blocking effect on apoptosis in HG-induced SV40 MES 13. Moreover, Rg3 also could ameliorate renal injury and weaken apoptosis of kidney tissues in diabetic model mice. Thus, we suggested that Rg3 has a significant protective effect on DKD.

Whether Rg3 can play a protective role on DKD by regulating miR-216a-5p has not been reported. In the current study, we confirmed that Rg3 could accelerate proliferation and restrain apoptosis by downregulating miR-216a-5p in HG-induced SV40 MES 13. Meanwhile, similar to Rg3, miR-216a-5p inhibition also could improve the pathological damage of the kidney and prevent apoptosis of kidney tissues in diabetic model mice. Thus, we also proved that miR-216a-5p inhibition also has a protective effect on DKD.

The occurrence of DKD involves diverse and complex cellular pathways, such as the MAPK pathway [1]. MAPK is a vital member of the MAPK family. High glucose can induce the activation of MAPK [39, 40]. Research showed that MAPK can promote cell proliferation, cause renal hypertrophy, glomerulosclerosis [41]. In recent years, researches also proved that some Chinese herbs (such as chlorogenic acid, apigenin, Huangkui capsule) have inhibitory effects on MAPK activity and can effectively reduce or even prevent the occurrence of DKD [42–44]. Studies also demonstrated that Rg3 can induce the activation of the MAPK pathway [28, 30]. In our study, we also revealed that Rg3 could activate the MAPK pathway by downregulating miR-216a-5p in HG-induced SV40 MES 13, and Rg3 or miR-216a-5p inhibition also could induce the MAPK pathway in kidney tissues of diabetic model mice. We initially revealed that Rg3 could prevent the development of DKD by downregulating miR-216a-5p and activating MAPK pathway. However, the specific mechanism still needs further verification.

In summary, ginsenoside Rg3 can suppress the apoptosis of renal tissue cells, leading to renal injury in DKD mice, and its mechanism may be relevant to the inhibition of miR-216a-5p.
